# Greater incidence of depression with hypnotic use than with placebo

**DOI:** 10.1186/1471-244X-7-42

**Published:** 2007-08-21

**Authors:** Daniel F Kripke

**Affiliations:** 1 The Scripps Clinic Sleep Center, 10666 North Torrey Pines Road, La Jolla, California 92037, USA; 2 Department of Psychiatry, Mail Code 0667, UCSD, 9500 Gilman Drive, La Jolla, California 92093-0667, USA

## Abstract

**Background:**

Although it has been claimed that insomnia causes an increased risk for depression, adequate controlled trials testing this hypothesis have not been available. This study contrasted the incidence of depression among subjects receiving hypnotics in randomized controlled trials versus those receiving placebo.

**Methods:**

The incidence of depression among patients randomized to hypnotic drugs or placebo was compiled from prescribing information approved by the United States Food and Drug Administration (FDA) and from FDA New Drug Application documents. Available data for zolpidem, zaleplon, eszopiclone, and ramelteon were accessed.

**Results:**

Data for 5535 patients randomized to a hypnotic and for 2318 randomized to placebo were compiled. The incidence of depression was 2.0% among participants randomized to hypnotics as compared to 0.9% among those randomized in parallel to placebo (p < 0.002).

**Conclusion:**

Modern hypnotics were associated with an increased incidence of depression in data released by the FDA. This suggests that when there is a risk of depression, hypnotics may be contra-indicated. Preventive treatments such as antidepressant drugs, cognitive-behavioral therapy, or bright light might be preferred. Limitations in the FDA data prevented a formal meta-analysis, and there was a lack of information about drop-out rates and definitions of depression. Trials specifically designed to detect incident depression when treating insomnia with hypnotic drugs and better summarization of adverse events in trials submitted to the FDA are both necessary.

## Background

There has been interest in the observation that insomnia predicts the incidence of major depressive disorders [1,2]. From the idea that the presence of insomnia predicts an increased risk for depression, some have theorized that insomnia is a cause of depression, implying that insomnia treatment might prevent depression.

The fact that insomnia predicts depression may simply be a tautological result of our definition of depression. According to DSM-IV [3], there are 9 groups of signs and symptoms which indicate a major depressive disorder. One of these is insomnia or hypersomnia. If 5 of these signs and symptoms are present, a diagnosis of major depressive disorder should usually be made. Since major depressive disorders often develop gradually as the number of symptoms increases, several of the 9 groups of symptoms are likely to precede the threshold when at least 5 are present. All of the 9 groups of signs and symptoms of major depression may be premonitory, and all predict the future onset of the full syndrome. Of the signs and symptoms of depression, insomnia is not the symptom with the highest risk factor or predictivity [4-6]. This indicates that insomnia could be a premonitory sign of a risk of depression but not a cause of major depression, any more than are the other signs and symptoms which combined, compose the definition of depression. Indeed, an association of insomnia with future depression may not prove that insomnia causes depression any more than epigastric pain causes peptic ulcer.

A longitudinal follow-up of insomnia, depression, and anxiety over time recently observed that insomnia did not predict future depression a decade later, independent of prior depression and anxiety, but use of hypnotics did [7]. This raises the interesting possibility that it might not be insomnia itself, but rather the associated usage of hypnotics, which increases the risk of future depression.

To determine whether insomnia itself causes depression, which might be prevented by use of hypnotics, or whether hypnotics might rather cause depression, randomized trials would be essential to demonstrate causality.

It happens that to examine long-term effects of modern hypnotics, pharmaceutical manufacturers have recently completed randomized trials of unprecedented size and duration. For the 4 most recently-approved drugs, the trials submitted by the industry in New Drug Applications (NDA) to the United States Food and Drug Administration (FDA) are now summarized in data which the FDA has provided on-line [8]. From the listings of adverse events in controlled trials, it is now possible to explore whether the most popular intervention to eliminate insomnia, the prescription of hypnotic drugs, reduces the incidence of depression.

## Methods

The on-line FDA NDA files for zolpidem, zaleplon, eszopiclone, and ramelteon consist of hundreds of pages of reports, data, interpretations, and correspondence concerning each drug [8]. The text of approved official "Prescribing Information" for each drug was also included. The files were searched for as much controlled trial information as was available. For specific sources of the data, see Additional file 1. The quality of the data did not make formal meta-analysis possible. Comparable data for other hypnotics approved earlier were not available [8].

From this information for each drug, the author extracted all summaries of parallel randomizing placebo-controlled trials (or combinations of trials) where the number of subjects studied in each group and the number suffering the adverse effect of depression were reported. Most of the "depression" adverse events tabulated may have been incident major depressions, but NDA data did not make clear if that was so. For most of these studies of healthy subjects and insomnia patients, participants with major depression at baseline were probably excluded, though details of exclusion criteria for each trial were often not available. The author then summed for the groups given each hypnotic and the comparable groups randomized to placebo, the total number of participants and the number of reports of incident depression (Table 1). In general, information on the definition of depression as an adverse effect, the type and severity of depression, its method of ascertainment, and the expertise of the dozens or hundreds of investigators performing these trials was not available. Dropout rates and the duration of hypnotic treatment before depression was recognized were not generally available.

**Table 1:** Depression incidence for four hypnotics and parallel placebos

**Drug**	**N subjects**	**Depression incidence**
Zolpidem	353	6
Zolpidem placebo	377	2
Eszopiclone	802	32
Eszopiclone placebo	294	3
Zaleplon	786	24
Zaleplon placebo	277	6
Ramelteon	3594	48
Ramelteon placebo	1370	11
**Total of 4 hypnotics**	5535*	110 (2.0%)*
**Total of 4 placebo groups**	2318*	22 (0.9%)*

*Chi Square = 10.04, p < 0.002, risk ratio = 2.1

Because the author has not received responses to some other queries to hypnotics manufacturers, no attempt was made to obtain additional data on these trials from the manufacturers. It was presumed that the data presented by the FDA are unbiased, though in many respects incomplete.

## Results

The sum of these compilations for the 4 hypnotics is provided in Table 1. Because several doses of the investigational hypnotics were compared to a placebo in some randomizing trials, there were more participants receiving hypnotics than parallel placebo. For each of the 4 hypnotics, the rate of incident depression was higher among participants randomized to hypnotic drugs (2.0%) than among contrast controls randomized to placebo (0.9%). Assignment to hypnotics was significantly associated with incident depression (Chi-Square = 10.04, p < 0.002). For incident depression, the risk ratio comparing hypnotics with placebo was 2.1 (95% Confidence Interval 1.3–3.3).

## Discussion

These data indicate that in randomized trials, depression was reported among participants receiving modern hypnotics significantly more often than among participants randomized to receive placebo. A possible interpretation would be that hypnotics are more likely to cause depression than to prevent it.

This is a post-hoc analysis of trials which were not designed primarily to examine depression. The compilation had many limitations which have caused some observers to doubt that causality has been demonstrated. Information limitations included trial details, the length of exposure of many participants (correcting for dropouts), and inadequate specification of the nature and severity of incident depressions. The quality of ascertainment of depression occurring as an adverse event was quite uncertain. It is not evident that a major depressive disorder was always diagnosed by an expert when depression was listed as an adverse event. There are potential statistical pitfalls in compiling results of numerous trials of different design and duration using 4 different hypnotics. Because the FDA online files are a limited source, other methods of ascertainment might have uncovered more trials of these drugs, especially post-marketing trials. The data utilized did not lend themselves to the techniques of formal meta-analysis. Many limitations of this compilation could not be overcome unless new trials with thousands of participants are done, so some uncertainty as to the present conclusions is unavoidable.

If drop-out rates were far higher in placebo than hypnotic groups, a greater incidence of depression in hypnotic groups might be attributable to longer exposure, but the study of Krystal et al. did not report an important difference in overall drop-out rates [9]. Notably, Krystal et al. reported a dropout rate of 2% due to depression in the eszopiclone group (presumably 12 participants) and 0% in the placebo group (Chi Square = 3.9, P < 0.05, my computation). Krystal et al. did not describe all 30 incident depressions listed in the FDA data for the same study (see Additional file 1 and [9].) One might suppose that dropouts had depression of sufficient severity to meet criteria for major depressive disorders, but depressions which did not cause dropout may have been milder. Dropout rates did not accompany incident depression rates in FDA data for the other studies.

Supporting the hypothesis that hypnotics cause depression is the statistical robustness of the results and the consistency of the subsamples (see Additional file 1), which all revealed more incident depression in hypnotic than placebo groups. Though the trial data analysed were unclear about the severity of the depressions which were associated with hypnotic use, a severe risk has been suggested by epidemiologic data, which found that the suicide rate was markedly elevated among hypnotic users at a time before the drugs listed in Table 1 came on the market [10,11]. It is also known that overall mortality has been elevated among hypnotics users, though it is unlikely that this increase in overall mortality would be largely attributable to depression [12]. Since the epidemiologic association data for the earlier drugs may be less indicative of causality than randomized trial results, it is unfortunate that the FDA web site did not report clinical trial data for the benzodiazepines popular in the 1980's, such as temazepam, flurazepam, and triazolam.

Many patients believe that better sleep can relieve depression, but sleep deprivation is known to be a powerful antidepressant treatment [13]. It is currently unknown if small increments in sleep, such as those produced by hypnotics, would tend to cause or to relieve depression. A recent meta-analysis raised a question whether newer nonbenzodiazepine hypnotics might increase total sleep time only a negligible amount [14]. Currently under investigation is whether cognitive-behavioral therapy (CBT) for insomnia, which often includes sleep restriction, will prove to be antidepressant [15-18]. A form of CBT which did not emphasize sleep restriction has been shown to improve sleep quality and general mental health [19,20].

One recent manufacturer-sponsored study might appear somewhat in conflict with these results [21]. Fava and colleagues reported that symptoms of major depression were reduced when eszopiclone was added to fluoxetine treatment. Considering reduction in Hamilton Depression Scores (with insomnia items removed) after 4 weeks, the effect of eszopiclone was not significant, but after 8 weeks, the improvement in depression scores was 13% greater with eszopiclone than with fluoxetine alone (p = 0.04). These results referred not to preventing the risk of major depression but to treating depression with fluoxetine once depression with related insomnia had appeared. Fluoxetine would be a poor choice of antidepressant among patients selected for insomnia symptoms, since fluoxetine often reduces sleep efficiency when an alternative such as trazodone would increase sleep efficiency [22]. This might be an example of a post-marketing trial designed to make the manufacturer's product appear favourable [23]. In any case, the finding of Fava et al. involved fewer participants and was less significant than the data in Table 1.

Another industry-supported study compared zolpidem 10 mg. vs. placebo in patients receiving one of 3 SSRI antidepressants (groups of 95 and 97 respectively) [24]. There were no significant differences between zolpidem and placebo in the non-sleep items of the Hamilton Depression Rating Scale. Similar to results of Krystal et al., there were two dropouts due to depression and one due to mania in the zolpidem group but none in the placebo group [24].

Another manufacturer-supported trial found that menopausal women randomized to eszopiclone had, on average, a significant improvement in mood compared with those who received placebo; however, the full ANCOVA statistics were not reported nor were the adjusted mean improvements [25]. One can infer that the difference between eszopiclone and placebo was only about 1 point on a small self-report scale which might confound sleep and mood. Any improvement might have occurred only in sleep. The mood result was reported as P < .05, which would not be significant after correction for multiple testing. This study reported no incident major depressions in either the hypnotic or placebo group.

In summary, the published literature partly supports and partly seems to contradict the compiled FDA data suggesting that hypnotics trigger depression. It is possible that hypnotics produce little effect on the median mood score but an increased the incidence of problematic exacerbations of depression.

## Conclusion

The quality of data which can be gleaned from published studies and unpublished NDA data available from FDA is not fully satisfactory, but manufacturer-sponsored trials show that hypnotics may be contra-indicated when insomnia signals a risk of depression developing. When premonitory insomnia occurs, preventive treatments such as antidepressant drugs, depression-oriented cognitive-behavioral therapy, and bright light treatment may be preferred.

## Competing interests

Dr. Kripke has no competing financial interests. Several years ago, Apollo Health, a light box manufacturer, donated some light boxes to his laboratory for research use. Dr. Kripke has published several studies concerning the risks of hypnotic drugs and is the author of a non-profit web site discouraging use of sleeping pills [26].

## Authors' contributions

DFK conceived, researched, and wrote this manuscript.

## Pre-publication history

The pre-publication history for this paper can be accessed here:



## Supplementary Material

Additional file 1 Sources of depression incidence data. Each web source is specified and details provided when available. Click here for file
