# Supplementary material for: Greater incidence of depression with hypnotic use than with placebo
Source: BMC Psychiatry. 2007 Aug 21;7:42. doi: 10.1186/1471-244X-7-42 (PMC1994947; doi:10.1186/1471-244X-7-42)
Supplement: Additional file 1 — Sources of depression incidence data. Each web source is specified and details provided when available. [file 1471-244X-7-42-S1.doc]

**Additional file 1 – Sources of depression incidence data**

| **Source** | **Sponsor** | **Doses, N's** | **P.I.** | **Locations** | **Duration** | **Drop-outs** | **Inclusion /Exclusion** |
| --- | --- | --- | --- | --- | --- | --- | --- |
| Derived from labeling information, Table 1, accessed July 10, 2007 [1] | Sanofi-Synthelabo | Zolpidem tartrate extended-release 12.5 mg: N=102 (depression N=2).  placebo: N=110 (depression N=0) | Not stated | Not stated | 3 weeks | Not stated | Not stated |
| Derived from labeling information, Table 2, accessed July 10, 2007 [1] | Sanofi-Synthelabo | Zolpidem tartrate extended-release 6.25 mg: N=99 (depressed mood N=1)  placebo: N=106 (depressed mood N=0) | Not stated | Not stated | 3 weeks | Not stated | Not stated |
| Derived from labeling information Table entitled Incidence of Treatment-Emergent Adverse Experiences in Long-term Placebo-Controlled Clinical Trials, accessed July 10, 2007 [2] | Sanofi-Aventis | Zolpidem 5-10 mg. N=152 (depression = 2%, i.e., probably N=3)  placebo N=161 (depression = 1%, probably N=2) | Not stated | Not stated | 28-35 nights | Not stated | Not stated |
| Derived from FDA medical review Table 8.1.5.3.1, accessed July 16, 2007 [3] | Wyeth-Ayerst | Zaleplon 5, 10, or 20 mg. N=786 (Depression 3%, presumably N=24)  Placebo N=277 (Depression 2%, presumably N=6) | Not stated | Not stated | 28 days | Not stated | Not stated |
| Derived from labeling information, Table 1, accessed July 16, 2007 [4] | Sepracor | Eszopiclone 2 mg. N=104 (N=4 depressed) eszopiclone 3 mg. N=105 (N=1 depressed)  placebo N=99 (N=0 depressed) | Not stated | Not stated | 6 weeks | not stated | not stated |
| Derived from FDA medical review Table H6, accessed July 16, 2007 [5] | Sepracor | Eszopiclone 3 mg. N=593 (N=27 depressions)  placebo N=195 (N=3 depressions) | Not stated[[1]](#footnote-2) | Not stated | 6 mo. | Not stated[[2]](#footnote-3) | Not stated[[3]](#footnote-4) |
| Derived from FDA medical review Table 29, accessed July 16, 2007 [6] | Takeda | Ramelteon <4 to 64 mg N=3594 (N=48 depressions)  placebo N=1370 (N=11 depressions)[[4]](#footnote-5) | Not stated | Not stated | Not stated | Not stated | Not stated |

1. Appears to be the same as: Krystal AD, Walsh JK, Laska E et al. Sustained efficacy of eszopiclone over 6 months of nightly treatment: results of a randomized, double-blind, placebo-controlled study in adults with chronic insomnia. Sleep 2003;26(7):793-9. [↑](#footnote-ref-2)
2. Krystal et al. stated that 360 (60.5%) of eszopiclone patients and 111 (56.6%) of placebo patients completed the study. Discontinuation due to depression was 2% in the eszopiclone group and 0% in the placebo group. [↑](#footnote-ref-3)
3. According to Krystal et al., "Patients receiving a DSM IV diagnosis of primary insomnia and reporting a usual total sleep time less than 6.5 hours per night and/or a usual sleep latency of more than 30 minutes each night for at least 1 month prior to screening were eligible for randomization, provided they did not (1) meet criteria for a DSM-IV Axis I psychiatric diagnosis other than primary insomnia, sexual and gender-identity disorders, or Axis II personality disorders (excluded by medical history); (2) have a history of substance abuse or substance dependence; (3) consume more than 2 alcoholic beverages per day or more than 14 per week; (4) use any psychotropic, hypnotic, or other medications known to affect sleep or to be contraindicated for use with hypnotics; or (5) use over-the counter analgesics that contain caffeine or herbal supplements, including products with herbs, melatonin, or St. John’s Wort." [↑](#footnote-ref-4)
4. A more limited subset were summarized in Table 6.1 of the document, in which the risk ratio for ramelteon was higher and significant (P<0.05). [↑](#footnote-ref-5)
